# A case control study on the relationship between occupational stress and genetic polymorphism and dyslipidemia in coal miners

**DOI:** 10.1038/s41598-023-29491-2

**Published:** 2023-02-09

**Authors:** Yongzhong Yang, Ziwei Zheng, Yuanyu Chen, Xuelin Wang, Hui Wang, Zhikang Si, Rui Meng, Jianhui Wu

**Affiliations:** 1 School of Public Health, North China University of Science and Technology, No. 21 Bohai Avenue, Caofeidian New Town, Tangshan, Hebei 063210 People’s Republic of China; 2 Hebei Province Key Laboratory of Occupational Health and Safety for Coal Industry, North China University of Science and Technology, Tangshan, Hebei People’s Republic of China

**Keywords:** Genetics, Psychology, Health occupations, Risk factors

## Abstract

Dyslipidemia is one of the known risk factors for cardiovascular disease, and its prevalence is increasing worldwide. At present, the study of dyslipidemia has gradually shifted from simple environmental or genetic factors to environment-gene interactions. In order to further explore the etiology and mechanism of dyslipidemia, we used occupational stress(OS) and *LYPLAL1*, *APOC3* and *SOD2* gene as research variables to explore their association with dyslipidemia.Here we used a case-control study to include Han workers from a coal mining enterprise in China to determine the association between study variables and dyslipidemia. Monofactor analysis showed that smoking, drinking, physical activity level, DASH diet score, sleep quality, BMI, hypertension, hyperuricemia, shift work, OS were significantly different between the two groups (*P* < 0.05). In the *APOC3* rs2854116 dominant model, patients with CT/CC genotype had a higher risk of dyslipidemia than those with TT genotype. In *SOD2* rs4880 recessive model, patients with GG genotype had a lower risk of dyslipidemia than those with AA/AG genotype, and the difference was statistically significant. We found that rs12137855 and OS, rs2854116 and OS, rs4880 and OS had joint effects, but no interaction based on the multiplication and addition model was found (*P*_*interaction*_ > 0.05). GMDR model showed that the rs12137855-rs2854116-rs4880-OS four-factor model had the highest cross-validation consistency and training-validation accuracy (*P* < 0.05), suggesting that there was a high-order interaction between them associated with dyslipidemia. We found that dyslipidemia in coal miners was related to OS and genetic factors. Through this study, we revealed the dual regulation of environmental factors and genetic factors on dyslipidemia. At the same time, this study provides clues for understanding the etiology and mechanism of dyslipidemia.

## Introduction

Metabolic syndrome is a global public health problem, and atherosclerosis is one of its typical symptoms. According to statistics, 80% of atherosclerosis is caused by dyslipidemia^1,2^. And atherosclerosis is closely related to the development of cardiovascular diseases^3^. The prevalence of dyslipidemia in Chinese adults has been increasing year by year^4^, and this phenomenon deserves attention and in-depth study.

Studies have shown an association between the level of lipid metabolism in the body and psychological stress^5^. According to statistics, more than 60% of the American professional believes that the main source of stress is work, and more than 40% of people feel stressed and anxious at work^6^. In addition to age, gender, and environmental factors such as smoking, alcohol, diet, and mental health^5,7–9^, the body's lipid levels are also regulated by genetic factors^10^: the *LYPLAL1* gene regulates the *LYPLAL1* in adipose tissue in the same way as triglyceride lipase^11,12^; *APOC3* inhibits lipoprotein lipase, hepatic lipase, and promotes the uptake of triglyceride (TG)^13^; manganese superoxide dismutase (Mn-SOD) is a scavenger of free radicals in the body, which can defend against free radicals attacking unsaturated fatty acids in biofilms and prevent lipid peroxidation^14^. All these genetic factors influence the catabolism and metabolism of body lipids by some mechanism.

Coal miners are more prone to occupational stress (OS) due to their prolonged exposure to a fast-paced and closed working environment^15^. Together with the presence of certain other specific occupational factors, they have a higher prevalence of dyslipidemia than the general population^16^. At present, we have not found any reports of association between OS and lipid-related genes. The Genome-Wide Association Study has reported that only a small proportion of dyslipidemia can be explained by lipid metabolism-related loci^17–19^. The development of dyslipidemia is usually the result of the interplay of multiple genes and factors, and most of the current studies only focus on environmental or genetic factors, while few studies have reported the effects of OS and gene interactions on dyslipidemia. Therefore, in this study, we selected rs12137855 in the *LYPLAL1* gene, rs2854116 in the *APOC3* gene and rs4880 in the *SOD2* gene to investigate the association of three gene polymorphism, OS and their interaction with dyslipidemia. To provide clues for an in-depth understanding of the etiology and mechanism of dyslipidemia.

## Methods

### Study design and participants

We used a case–control study design. Cases were selected from 445 Chinese Han miners who participated in occupational health checkups diagnosed with dyslipidemia in a Chinese coal mine enterprise from July to September 2020. Inclusion criteria for the case group: ① those with dyslipidemia; ② length of service ≥ 1 year; exclusion criteria: ① those with diabetes, severe cardiovascular disease, chronic hepatitis or cirrhosis, or malignant tumors; ② those with incomplete questionnaires; ③ those without blood samples. Inclusion criteria for the control group: ① non-dyslipidemia coal miners who participated in the physical examination during the same period; ② length of service ≥ 1 year^20^. Exclusion criteria were the same as those for the case group. Two groups were matched according to age and gender, and 445 healthy controls were finally included. The study was approved by the Ethics Committee of North China University of Science and Technology (No. 15006), and all study subjects had given informed consent.

### Survey content

Our investigators were strictly and uniformly trained to conduct a one-on-one questionnaire survey of coal miners to collect the following information: ① age, gender, length of service, education level, marital status, monthly household income, smoking and alcohol consumption, eating behavior, sleep quality (used Athens Insomnia Scale^21^), physical activity level, and shift work; ② OS: used the Chinese version of the Job Content Questionnaire (JCQ) developed by Yan Sha et al. (Cronbach 's α = 0. 74)^22^; ③ physical examination: height, weight, blood pressure. After participants fasted for 12 h, venous blood was drawn from 8:00 to 9:30 am the next day, and TG, total cholesterol (TC), low density lipoprotein-cholesterol (LDL-C), and high density lipoprotein-cholesterol (HDL-C) were measured by the autoanalyzer (type BS-400; Mindray Bio-Medical Electronics Co., Ltd., Shenzhen, China). Serum TC, TG, HDL-C, and LDL-C levels were determined using commercially available enzymatic assay kits.

### Variables explanation

The diagnostic criteria for dyslipidemia in this study were meeting one of TC ≥ 6.2 mmol/L, TG ≥ 2.3 mmol/L, LDL-C ≥ 4.1 mmol/L, HDL-C < 1.0 mmol/L or being treated after diagnosis^23^. The JCQ covers 3 dimensions (5 items), job autonomy (9 items), and social support (8 items), with a total of 22 items. OS was evaluated by using the Likert 4-point scale, and each item was scored from 1 to 4. OS was evaluated by using the job demand-control ratio (D/C ratio), which was determined as OS if the D/C ratio was ≥ 1 and non-OS if the D/C ratio was < 1. The higher the D/C ratio, the higher the degree of OS.

$$\mathrm{D}/\mathrm{C ratio}=\frac{Score\,of\,job\,demand}{Score\,of\,job\,control}\times \frac{9}{5}$$

Smoking was defined as ≥ 1 cigarette per day and ≥ 6 months of continuous smoking. Drinking was defined as drinking at least 2 times per week with at least 50 g of ethanol intake per occasion for ≥ 1 year. Dietary behavior was assigned according to the Dietary Approaches to Stop Hypertension (DASH) model^24^. Physical activity levels were grouped using the International Physical Activity Questionnaire assignment criteria^25^. Body Mass Index (BMI) was the ratio of body weight (kg) to height squared (m^2^). Hypertension was defined as a current systolic blood pressure ≥ 140 mmHg or diastolic blood pressure ≥ 90 mmHg or being treated with antihypertensive therapy. Hyperuricemia (HUA) was defined as a uric acid level ≥ 420 μmol/L in men and ≥ 357 μmol/L in women^26^.

### Genomic DNA extraction and typing

In this study, the genomic DNA was extracted using the DNA extraction kit from Tiangen Biochemical Technology Co. We used Sequenom Genotyping Tools and Mass ARRAY Assay Design 3.1 software to design polymerase chain reaction (PCR) amplification primers and single-base extension primers for the single nucleotide polymorphism (SNP) to be tested. Gene polymorphism was detected by Polymerase Chain Reaction-Restriction Fragment Length Polymorphism Technique. See Table 1 for SNP loci information.

**Table 1:** SNP loci information.

RS	Chr	Position	Ref	Alt	Gene	Region	1000G_CHBS	1000G_EAS
rs12137855	1	219448378	C	T	LYPLAL1	Intergenic	0.070	0.079
rs2854116	11	116700169	C	T	APOC3	Upstream	0.546	0.530
rs4880	6	160113872	A	G	SOD2	Exonic	0.113	0.125

### Statistical methods

Data for measures were described and compared between groups using x ± s and t test when the data obeyed normal distribution, otherwise M (P_25_, P_75_) and rank sum test were used. Statistical data were expressed as rates or composition ratios and compared between groups using Pearson x^2^ test. For all SNP loci, the Hardy–Weinberg equilibrium (HWE) test was used to determine whether the control study subjects were a random sample of the target population. The association between genetic models and dyslipidemia abnormalities was analyzed using SNPStats online software (https://www.snpstats.net/start.htm.), and Akaike Information Criterion (AIC) and Bayesian Information Criterion (BIC) were used for optimal model selection. Andersson additive interaction model^27^ and logistic regression multiplicative interaction model were used to explore the interaction between the three genes and OS, and Generalized-Multifactor-Dimensionality-Reduction (GMDR) model was used to analyze the existence of gene–gene and gene-OS interactions with dyslipidemia. The test level α = 0.05 (both sides). The above statistical analysis was processed and analyzed using IBM SPSS 23.0 and GMDR 0.9 software.

### Ethics approval and consent to participate

All procedures performed in studies involving human participants were in accordance with the ethical standards of the institutional and/or national research committee and with the 1964 Helsinki declaration and its later amendments or comparable ethical standards. The study was approved by the Ethics Committee of North China University of Science and Technology (NO.15006). All individuals in the study signed a paper version of the informed consent.

## Results

### Comparison of the basic characteristics and health status of the two groups

The mean age of the study subjects included in this study was 40.24 ± 7.47 years. There were 850 males and 40 females. The basic characteristics and physical health status of the study subjects were compared between the two groups, and we found that the differences in smoking, drinking, physical activity level, DASH diet score, BMI, hypertension, shift work, sleep quality, HUA, and OS were statistically significant in the case and control groups (*P* < 0.05). The differences in age, gender, length of service, education level, marital status, and monthly household income of the study subjects were not statistically significant in the two groups. As shown in Table 2 (at the end of the article).

**Table 2:** Comparison of basic characteristics and physical health status between the case group and the control group [n(%)].

Factors	Control group (n = 445)	Case group (n = 445)	*χ*^2^	*P*
Age				
< 30	23 (5.1)	27 (6.1)	0.007^a^	0.93
30 ~	222 (49.8)	217 (48.8)		
40 ~	138 (31.1)	134 (30.1)		
50 ~	62 (14.0)	67 (15.0)		
Gender				
Females	20 (4.4)	17 (3.8)	0.25	0.61
Males	425 (95.6)	428 (96.2)		
Length of service				
< 5	23 (5.1)	14 (3.2)	2.406^a^	0.12
5 ~	69 (15.5)	61 (13.8)		
10 ~	130 (29.2)	133 (29.8)		
15 ~	223 (50.2)	237 (53.2)		
Education				
Elementary school and below	28 (6.2)	24 (5.4)	0.72	0.70
High school/technical secondary school	298 (66.9)	309 (69.4)		
College and above	119 (26.9)	112 (21.2)		
Marital status				
Unmarried	9 (2.0)	12 (2.7)	0.41	0.52
Married	436 (98.0)	438 (97.3)		
Monthly household income (yuan)				
< 5000	81 (18.2)	111 (24.9)	1.90^a^	0.17
5000 ~	131 (29.5)	119 (26.7)		
6000 ~	92 (20.6)	73 (16.5)		
7000 ~	141 (31.7)	142 (31.9)		
Smoking status^b^				
No smoking	188 (42.2)	228 (51.2)	7.22	0.007
Quit smoking or smoking	257 (57.8)	217 (48.8)		
Drinking status^c^				
No drinking	140 (31.4)	177 (39.8)	6.71	0.01
Alcohol withdrawal or drinking	305 (68.6)	268 (60.2)		
Physical activity level				
Mild	179 (40.2)	46 (10.3)	110.08^a^	< 0.001
Moderate	49 (11.0)	45 (10.1)		
Severe	217 (48.8)	354 (79.6)		
DASH diet Score				
< 20	171 (38.4)	109 (24.5)	10.49^a^	0.001
20 ~	98 (22.0)	122 (27.4)		
22 ~	79 (17.8)	106 (23.8)		
24 ~	97 (21.8)	108 (24.3)		
Sleep quality				
No insomnia	409 (91.9)	385 (86.5)	7.75	0.02
Suspected insomnia	29 (6.5)	53 (11.9)		
Insomnia	7 (1.6)	7 (1.6)		
BMI (kg/m^2^)				
< 24	152 (34.2)	105 (23.6)	8.80^a^	0.003
24 ~	190 (42.6)	219 (49.2)		
28 ~	103 (23.2)	121 (27.2)		
Hypertension				
No	166 (37.3)	196 (44.0)	2.86	0.09
Yes	279 (62.7)	249 (56.0)		
HUA				
No	380 (85.3)	346 (77.7)	8.64	0.003
Yes	65 (14.7)	99 (22.3)		
Shift work situation				
No	169 (38.0)	205 (40.1)	5.98	0.04
Yes	276 (62.0)	240 (59.9)		
OS				
No	169 (38.0)	227 (51.0)	15.31	< 0.001
Yes	276 (62.0)	218 (49.0)		

^a^Chi-square test of trend.

^b^The Quit smoking Group was smaller and was incorporated into the smoking group.

^c^The Alcohol withdrawal Group was smaller and was incorporated into the drinking group.

### The logistic regression analysis of OS and dyslipidemia

In the logistic regression analysis of OS and dyslipidemia, model 1 adjusted only for education, marital status, and monthly household income. The results showed that the risk of dyslipidemia in the OS group was 1.76 times higher than that in the non-OS group (OR  = 1.76, 95% CI 1.35–2.31). Model 2 further adjusted for lifestyle indicators based on model 1: smoking, drinking, physical activity level, DASH diet score, sleep quality, and shift work. We found an enhanced association between OS and dyslipidemia, with the risk of dyslipidemia in the OS group being 2.00 times higher than that in the non-OS group (OR  = 2.00, 95% CI 1.48–2.71). Model 3 further adjusted for health status indicators: BMI, hypertension, and HUA on the basis of model 2. The results were consistent with model 1 and model 2. The differences of all three models were statistically significant (*P* < 0.001). As shown in Table 3.

**Table 3:** Logistic regression analysis of occupational stress and risk of dyslipidemia.

Model	OS	OR (95% CI)	*P*
1^a^	No	1.00	–
	Yes	1.76 (1.35–2.31)	< 0.001
2^b^	No	1.00	–
	Yes	2.00 (1.48–2.71)	< 0.001
3^c^	No	1.00	–
	Yes	2.02 (1.49–2.75)	< 0.001

^a^The variables adjusted by model 1 were education, marital status and monthly family income.

^b^Model 2 further adjusted smoking status, drinking status, physical activity level, DASH diet score, sleep quality and shift work situation on the basis of model 1.

^c^Model 3 further adjusted BMI, hypertension, and HUA on the basis of model 2.

### Association of three genetic polymorphism with dyslipidemia

#### The HWE test for the control group

The HWE test results for the three gene polymorphic loci in the control group showed that the rs12137855 in the *LYPLAL1* and rs2854116 in the *APOC3* conformed to the HWE law (*P* > 0.05) and were well represented; but the rs4880 in the *SOD2* did not conform to the law. As shown in Table 4.

**Table 4:** The Hardy–Weinberg equilibrium test for the control group.

Gene	Site	Allele	*χ*^2^	*P*
LYPLAL1	rs12137855	C/T	1.26	0.26
APOC3	rs2854116	C/T	0.01	0.94
SOD2	rs4880	A/G	10.0	0.01

#### Association of gene polymorphism with dyslipidemia

Due to the low frequency of the rs12137855 in the *LYPLAL1* T allele in the population, mutant homozygous genotype TT at this locus was not observed in this study. Using wild homozygous type CC as the reference group, we did not find an association between the rs12137855 locus polymorphism and dyslipidemia in coal miners. However, in this analysis we found a higher risk of dyslipidemia in the co-dominant model of rs2854116 in the *APOC3* in those carrying the CC genotype than in those carrying the TT genotype (OR  = 1.59, 95% CI 1.03–2.45), but the test did not reach a significant level. In the dominant model, those carrying the CT/CC genotype had a statistically significant higher risk compared with those carrying the TT genotype (OR  = 1.39, 95% CI 1.01–1.91). The results of the co-dominant model of rs4880 in the *SOD2* showed that coal miners carrying the mutant homozygous type GG had a reduced risk of disease (OR  = 0.27, 95% CI 0.11–0.64), using the wild homozygous type AA as the reference group. In the recessive model, those carrying the GG genotype had a significantly lower risk of dyslipidemia compared to those carrying the AA/AG genotype (OR  = 0.28, 95% CI 0.12–0.67). The differences between the above models were statistically significant, and combining AIC and BIC, the recessive model was the optimal model among the four models. The results are shown in Table 5.

**Table 5:** Association analysis of each gene polymorphism and dyslipidemia (n[%]).

Model	Genotype	Controls	Dyslipidemia	OR (95% CI)	*P*	AIC	BIC
rs12137855							
	CC	400 (89.9)	393 (88.3)	1.00	0.30	1062.1	1143.5
	CT	45 (10.1)	52 (11.7)	1.29 (0.80–2.11)			
rs2854116							
Codominant	TT	158 (35.5)	131 (29.4)	1.00	0.088	1060.3	1140.5
	CT	215 (48.3)	215 (48.3)	1.32 (0.94–1.85)			
	CC	72 (16.2)	99 (22.2)	1.59 (1.03–2.45)			
Dominant	TT	158 (35.5)	131 (29.4)	1.00	0.044	1059.1	1140.5
	CT-CC	287 (64.5)	314 (70.6)	1.39 (1.01–1.91)			
Recessive	TT-CT	373 (83.8)	346 (77.8)	1.00	0.13	1060.8	1142.3
	CC	72 (16.2)	99 (22.2)	1.35 (0.92–1.98)			
Overdominant	TT-CC	230 (51.7)	230 (51.7)	1.00	0.49	1062.7	1144.1
	CT	215 (48.3)	215 (48.3)	1.11 (0.82–1.50)			
rs4880							
Codominant	AA	297 (66.7)	331 (74.4)	1.00	0.005	1054.7	1140.9
	AG	120 (27.0)	106 (23.8)	0.83 (0.58–1.17)			
	GG	28 (6.3)	8 (1.8)	0.27 (0.11–0.64)			
Dominant	AA	297 (66.7)	331 (74.4)	1.00	0.048	1059.3	1140.7
	AG-GG	148 (33.3)	114 (25.6)	0.72 (0.52–1.00)			
Recessive	AA-AG	417 (93.7)	437 (98.2)	1.00	0.002	1053.8	1135.3
	GG	28 (6.3)	8 (1.8)	0.28 (0.12–0.67)			
Overdominant	AA-GG	325 (73.0)	339 (76.2)	1.00	0.47	1062.6	1144.1
	AG	120 (27.0)	106 (23.8)	0.88 (0.62–1.24)			

Adjusted variables included: smoking status, drinking status, DASH diet score, physical activity level, sleep quality, BMI, hypertension, HUA, shift work situation, and OS.

### Association of OS-gene interactions with dyslipidemia

#### The multiplicative interaction of OS-gene

A cross-classification interaction analysis was performed between OS and various genotypes of the three genes. The group non-OS and CC genotype at the rs12137855 was used as the reference group, the results showed a reduced risk of dyslipidemia in those with OS and CC genotype (OR  = 0.50, 95% CI 0.36–0.69) after correcting for potential confounders. Using the non-OS group with TT genotype at rs2854116 as the reference group, the risk of dyslipidemia was reduced in OS with TT genotype (OR  = 0.40, 95% CI 0.23–0.68) and CT genotype (OR  = 0.60, 95% CI 0.37–0.97). Using non-OS and the rs4880 locus *AA* genotype as a reference, we found that the risk of dyslipidemia was reduced in miners with OS and carrying all three genotypes: AA genotype (OR  = 0.51, 95% CI 0.36–0.74), AG genotype (OR  = 0.45, 95% CI 0.27–0.73), and GG genotype (OR  = 0.11, 95% CI 0.04–0.37), and the strength of the association gradually increased. However, we did not find a multiplicative interaction between OS and *LYPLAL1*, *APOC3*, and *SOD2* (*P*_*interaction*_ > 0.05). The results are shown in Table 6.

**Table 6:** Analysis of the cross- classification interaction of OS-gene.

Genotype	OS		*P*_*interaction*_
	No	Yes	
rs12137855			
CC	1.00	0.50 (0.36–0.69)	0.62
CT	1.09 (0.50–2.39)	0.72 (0.39–1.34)	
rs2854116			
TT	1.00	0.40 (0.23–0.68)	0.34
CT	1.12 (0.67–1.89)	0.60 (0.37–0.97)	
CC	1.12 (0.59–2.11)	0.86 (0.46–1.61)	
rs4880			
AA	1.00	0.51 (0.36–0.74)	0.82
AG	0.78 (0.46–1.31)	0.45 (0.27–0.73)	
GG	0.34 (0.09–1.31)	0.11 (0.04–0.37)	

Adjusted variables included: Smoking status, drinking status, DASH diet score, physical activity level, sleep quality, BMI, hypertension, HUA, and shift work situation.

#### The additive interaction of OS-gene

The Andersson additive interaction model was used in this study to analyze the additive interaction of OS-gene. Since the dominant model at locus rs2854116 is the optimal model and locus rs4880 is the recessive model, we chose the corresponding model in further analysis. Using the Excel template provided by Andersson et al. the Relative Excess Risk of Interaction (RERI) and Attributable Proportion of Interaction (AP) were calculated and the results showed that the 95% CI for RERI all included 0. Therefore, it cannot yet be assumed that OS and the three genetic polymorphisms exist based on the additive model of interaction. The results are shown in Table 7.

**Table 7:** Analysis of the additive interaction of OS-gene.

OS-gene	RERI (95% CI)	AP (95% CI)
OS-rs12137855	0.098 (− 0.720 to 0.916)	0.130 (− 0.937 to 1.198)
OS-rs2854116	0.117 (− 0.587 to 0.821)	0.127 (− 0.614 to 0867)
OS-rs4880	0.235 (− 0.196 to 0.666)	1.554 (− 1.292 to 4.399)

#### GMDR model of gene–gene and os-gene interactions and dyslipidemia risk

To analyze the possible higher-order interactions among *LYPLAL1*, *APOC3*, and *SOD2*, the GMDR model was used to analyze the association between gene–gene interactions and dyslipidemia in coal miners. After adjusting for relevant variables, it was found that the three-factor (rs12137855-rs2854116-rs4880) model had high testing balance accuracy and cross-validation consistency, but the model test results did not reach a statistically significant level (*P* > 0.05), and the interaction between the three genes could not yet be considered to exist. See Table 8.

**Table 8:** Analysis of the GMDR model of gene–gene.

Model	Training balance accuracy	Testing balance accuracy	*P*	Cross-validation consistency
rs4880	0.5259	0.5120	0.377	8/10
rs2854116-rs4880	0.5290	0.5025	0.377	5/10
rs12137855-rs2854116-rs4880	0.5328	0.5240	0.172	10/10

Adjusted variables included: Smoking status, drinking status, DASH diet score, physical activity level, sleep quality, BMI, hypertension, HUA, shift work situation, and OS.

However, we analyzed the OS-gene interaction based on the GMDR model and found that the single-factor model (OS), the three-factor model (rs2854116-rs4880-OS) and the four-factor (rs12137855-rs2854116-rs4880-OS) model had the greatest cross-validation consistency, and the results of the three model tests were statistically significant. Since the testing balance accuracy of the four-factor model was the highest, the best model was the four-factor interaction model. As shown in Table 9.

**Table 9:** Analysis of the GMDR model of OS-gene.

Model	Training balance accuracy	Testing balance accuracy	*P*	Cross-validation consistency
OS	0.5802	0.5792	0.001	10/10
rs2854116-OS	0.5879	0.5747	0.001	7/10
rs2854116-rs4880-OS	0.5961	0.5954	0.001	10/10
rs12137855-rs2854116-rs4880-OS	0.5965	0.5810	0.001	10/10

Adjusted variables included: Smoking status, drinking status, DASH diet score, physical activity level, sleep quality, BMI, hypertension, HUA, and shift work situation.

## Discussion

With the continuous development and progress of society, the traditional medical model has changed. The focus of research on risk factors for chronic noncommunicable diseases is no longer limited to traditional factors, but is gradually including psychosocial factors as well. This is why OS has started to receive a lot of attention from researchers. In this study, OS was found to be a risk factor for dyslipidemia in coal miners by logistic regression analysis, which is consistent with the conclusions reached by some researchers^28,29^. The mechanism by which OS affects lipid metabolism has not been elucidated, and two hypotheses are mainly considered. One is that work stress affects the sympathetic-adrenal-medullary and hypothalamic–pituitary–adrenal axes of the body, which stimulates increased adrenaline secretion and leads to abnormal lipid metabolism^30^. The other is the indirect behavioral pathway, where being under prolonged stress may alter the individual's perception of need and can produce undesirable behavioral patterns such as smoking and drinking, which can lead to dyslipidemia. The health status of miners affects the national economy to a certain extent, so their physical and mental health should attract our attention.

The *LYPLAL1* gene encodes a protein with sequence similarity to human acyl protein thioesterasesl and is found in adipose tissue. It was initially widely studied as a genetic susceptibility gene for obesity and type II diabetes, and later researchers found that its polymorphism were also associated with atherosclerosis and nonalcoholic fatty liver^31–34^. This is due to the fact that *LYPLAL1* gene possesses triglyceride lipase activity, which may be involved in the process of inhibiting TG degradation, thus affecting lipolysis and metabolism in the body^11,12,35^. However, studies on the role of *LYPLAL1* gene in lipid metabolism are still controversial^36^. In this study, 890 coal miners were recruited, including 445 each from patients with dyslipidemia and healthy control populations. We analyzed the association of rs12137855 polymorphism with dyslipidemia, and no association was found between the findings. However, we performed cross-classification interaction analysis between OS and rs12137855 polymorphism and showed that the CC genotype of this locus in OS population reduced the risk of dyslipidemia, suggesting a combined effect between the two. Because the frequency of the T allele at this locus is low in the Chinese Han population and the TT genotype was not observed in the subjects included in this study, the association of the rs12137855 s polymorphism with dyslipidemia needs to be further investigated.

The *APOC3* gene belongs to the APO protein family and is mainly found in celiac particles and very low density lipoproteins (VLDL). It is involved in VLDL/LDL metabolism, inhibits lipoprotein esterase activity and reduces TG catabolism^37^. An animal model noted that TG levels were significantly increased in *APOC3* overexpressing transgenic mice^38^. A study on the association of rs2854116 locus polymorphism with metabolic syndrome found that the frequency of C allele was significantly higher in patients with metabolic syndrome than in non-metabolic syndrome patients, and that C allele carriers had increased TG and HDL-C levels compared to wild genotype carriers, which is similar to the results of our study^39^. The rs2854116 in the *APOC3* carries a significantly increased risk of CC/CT genotype compared with those carrying TT genotype, suggesting that the C allele of rs2854116 locus may be associated with the development of dyslipidemia. We further analyzed its interaction with OS and found that there was a combined effect of OS and rs2854116 locus: those with OS and TT/CT genotype had a lower risk of dyslipidemia than those with non-OS and TT genotype.

Mn-SOD can play an important role in maintaining the dynamic balance of superoxide anion radical production and elimination in the organism by scavenging superoxide anion radicals produced in vivo^14^. Recent studies have pointed out that rs4880 in the *SOD2* mutation can lead to reduced Mn-SOD activity and is closely associated with diabetes, atherosclerosis, coronary heart disease, and tumorigenesis^40^. However, there are few studies on its association with dyslipidemia, and the mechanism of which has not been elucidated. In our study, we found that rs4880 polymorphism was associated with dyslipidemia, and the risk of dyslipidemia was significantly lower in those carrying AG/GG genotype, suggesting that the G allele may be a protective factor for dyslipidemia. In the analysis of the interaction between OS and *SOD2*, it was found that those carrying all three genotypes in the OS group had a reduced risk of dyslipidemia compared to the non-OS group. It is suggested that there is a combined effect of rs4880 and OS. Since the distribution of the rs4880 in the control group of the present study subjects did not conform to the HWE test. Therefore, the population representation of this study is limited, and the association between rs4880 and dyslipidemia needs to be further investigated.

In addition, the results of the analysis of interactions in this study did not reveal any interaction between OS-rs12137855, OS-rs2854116, and OS-rs4880 based on multiplicative or additive models, but higher-order interactions between the three genes and OS were found by the GMDR model. Due to the specificity of the study population, the women included in this study and the sample size were small, resulting in a limited population representation. Also, this study only explored the relationship between the three genes and dyslipidemia and did not delve into the association between them and the components of dyslipidemia. Further studies on the relationship between other related genes, OS and dyslipidemia are needed.

## Conclusions

This study uses OS and genes (*LYPLAL1*, *APOC3* and *SOD2*) as research variables, and explores their association with dyslipidemia in coal workers from the perspective of the interaction between environment and genetics. The results show that dyslipidemia in coal workers was related to OS and genetic factors. Through this study, we revealed the dual regulation of environmental factors and genetic factors on dyslipidemia. At the same time, this study provides clues for understanding the etiology and mechanism of dyslipidemia.

## Data Availability

Data are available upon reasonable request. The datasets generated and analyzed during the current study are not publicly available due other analyses are proceeding but are available from the corresponding author on reasonable request.

## Abbreviations

OS: Occupational stress
TG: Triglyceride
Mn-SOD: Manganese superoxide dismutase
JCQ: The job content questionnaire
TC: Total cholesterol
LDL-C: Low density lipoprotein-cholesterol
HDL-C: High density lipoprotein-cholesterol
D/C ratio: The job demand-control ratio
DASH: The dietary approaches to stop hypertension
BMI: Body mass index
HUA: Hyperuricemia
PCR: Polymerase chain reaction
SNP: The single nucleotide polymorphism
HWE: The Hardy–Weinberg equilibrium
AIC: Akaike information criterion
BIC: Bayesian information criterion
GMDR: Generalized-multifactor-dimensionality-reduction
RERI: The relative excess risk of interaction
AP: Attributable proportion of interaction
VLDL: Very low density lipoproteins
